# Endothelial keratoplasty for Fuchs dystrophy

**Published:** 2017

**Authors:** Laura Macovei, Ioana Gobej

**Affiliations:** * Ophthalmology Department, “Dr. Carol Davila” Central University Military Emergency Hospital, Bucharest, Romania

**Keywords:** corneal graft, DMEK, Fuch’s endothelial dystrophy

## Abstract

We report the case of a 69-year-old female with Fuchs endothelial dystrophy and posterior chamber in-the-bag intraocular lens, whom we treated with DMEK surgical technique. We encountered difficulties both during obtaining the endothelium from the young donor and during the intraocular unrolling and its application on the stroma. We evaluated both preoperative and postoperative the following parameters: visual acuity, slit lamp, and optical coherence tomography appearance of the cornea, corneal thickness, intraocular pressure, endothelial cell density. In all postoperative assessments, the endothelium was attached to the host's stroma, with small peripheral, inferior folding area. Short-term results were favorable with a clear cornea and significant improvement in visual acuity obtained in the first 3 months of postoperative follow-up.

**Abbreviation**:

BCVA = best-corrected visual acuity, RE = right eye, LE = left eye, OCT = optical coherence tomography, DMEK = Descemet membrane endothelial keratoplasty

## Introduction

Great progress has been achieved lately in corneal surgery, especially in corneal grafting for endothelial failure. The once gold standard penetrating keratoplasty has been replaced by more advanced and superior techniques such as DSAEK (Descemet’s stripping automated endothelial keratoplasty), UT-DSAEK (ultra-thin DSAEK) and, most recently, DMEK [**1**]. DMEK is a partial-thickness corneal graft surgery in which only Descemet’s membrane and endothelium are replaced, unlike DSAEK and UT-DSAEK, in which the donor tissue includes a fine lamella of corneal stroma.

Today, numerous articles and studies show the superiority of DMEK versus DSAEK and UT-DSAEK in terms of BCVA, graft rejection [**2**,**3**], corneal topography [**4**,**5**], choice of patient [**6**]. Although still very used (for their more rapid learning curve), DSAEK and UT-DSAEK have lost a great part of their indications in the advantage of DMEK [**7**].

In the majority of cases, endothelial keratoplasty by DMEK is a method of choice for corneal pathologies with endothelial dysfunction [**8**] but also a challenge, because of both donor tissue preparation and its manipulation for positioning on the recipient's stroma.

## Material and methods – Case report

A 65-year-old woman presented in our clinic with gradual decreased vision for the past two years, more significant in the RE. The patient was diagnosed with Fuchs’ dystrophy 3 years prior to presentation and she had cataract surgery of the RE two years earlier. She was also complaining of foreign body sensation and, sometimes, eye pain. Past medical history of the patient was insignificant with no family history of eye diseases. General review of systems was negative.

At presentation, BCVA was: RE = 0.1, LE = 0.7; manifest refraction was RE: +1,5 dcyl 160, LE: +3 dsf +1,25 dcyl 176; intraocular pressure on non contact tonometry was RE: 26 mmHg, LE: 23 mmHg; pachymetry was RE: 675 µm, LE: 631 µm. Slit-lamp examination of the RE revealed corneal beaten metal aspect, confluent guttae, descemets folds, significant stromal edema, epithelial edema with small defect areas, posterior chamber intraocular lens implanted in the bag. LE presented endothelial beaten metal appearance, confluent guttae, stromal edema, cortical opacities, and mild nuclear sclerosis of the lens. Dilated fundus examination was impossible to perform at RE, retinal angiosclerosis being shown at LE.

Specular microscopy of the corneal endothelium of the LE revealed decreased cell density (1100/ mm²) with abnormal cell morphology (polymegathism and pleomorphism) and the presence of endothelial guttae. Specular microscopy was not possible to perform at the RE. The anterior segment OCT showed increased central corneal thickness in both eyes, posterior corneal surface irregular and highly reflective, prominent formations in anterior chamber, small, hyporeflective intrastromal spaces. 

The patient’s symptoms and examination were consistent with the diagnosis of Fuchs’ endothelial dystrophy in both eyes, complicated with bullous keratopathy in RE, cataract in LE, posterior chamber pseudophakia in RE.

Treatment options including medical therapy and surgical intervention were discussed with the patient and she elected surgery by Descemet’s membrane endothelial keratoplasty.

For the preparation of the donor tissue, we used the SCUBA (Submerged Cornea Using Backgrounds Away) technique (**Fig. 1 A**-**D**). We have centered the donor cornea with the endothelial side up on the punch block, we scored the peripheral cornea at the Schlemm’s canal level with a blunt instrument, we have gently stripped the endothelial tissue leaving a small adhesion in center, we have punched the donor with 8,5 mm trephine, lifted it from the stromal adhesion carefully using the “one touch” technique and stained it with trypan blue. The donor tissue, double rolled, was loaded into a glass injector by aspiration. The difficulty of the preparation was the younger age of the donor: the endothelial tissue was thin, prone to tears, and adherent to the corneal stroma.

**Fig. 1 A:**
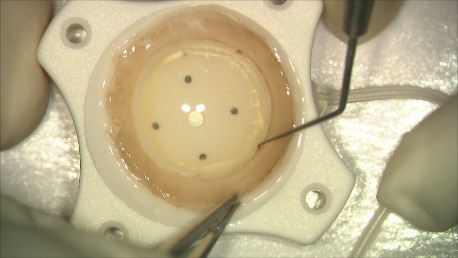
Scoring the donor’s endothelium

**Fig. 1 B:**
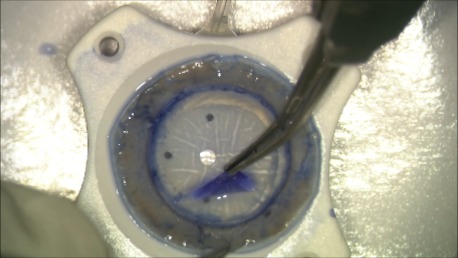
Partially stripping the endothelium after the coloration with trypan blue

**Fig. 1 C:**
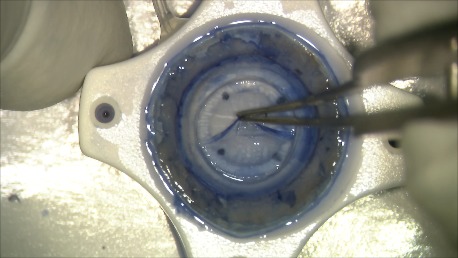
Finalizing the stripping of the endothelium

**Fig. 1 D:**
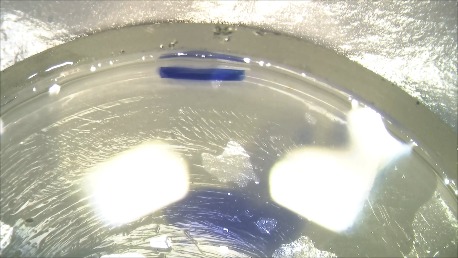
Double rolled donor tissue in BSS

The surgical technique we used for this patient was Descemet Membrane Endothelial Keratoplasty, with the following main steps (**Fig. 2 A**-**E**): performing retrobulbar anesthesia, desepitheliation for better visualization, marking the peripheral cornea at 9 mm diameter, creating 3 paracentesis, filling the anterior chamber with air, scoring and stripping Descemet’s membrane with reverse Sinskey hook following the peripheral markings, completing a descemetorhexis, keratotomy and removal of the Descemet membrane through the main wound, injecting acetylcholine intraocular solution into the anterior chamber to constrict the pupil, creating an inferior peripheral iridotomy, removing air and injecting BSS leaving a shallow anterior chamber, injecting the endothelium very carefully into the anterior chamber with double-roll up, suturing the wound with 10-0 nylon. Unscrolling and orientating the DMEK-graft using different “no-touch” techniques were very difficult due to the young donor’s age. The graft was properly positioned and centered on the recipient’s stroma but the persistence of peripheral folds could not be avoided. The graft position was secured by filling the anterior chamber with air and positioning the patient supine as much as possible until complete air resorption. 

**Fig. 2 A:**
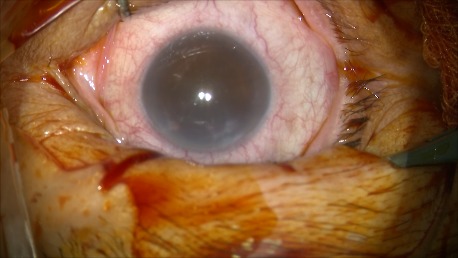
Cornea at the moment of surgery

**Fig. 2 B:**
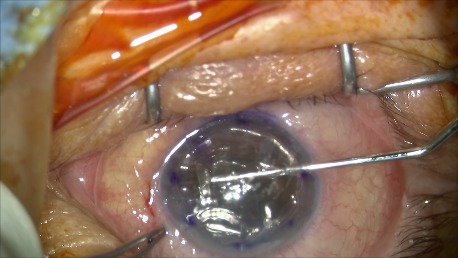
Descemetorhexis after marking the cornea

**Fig. 2 C:**
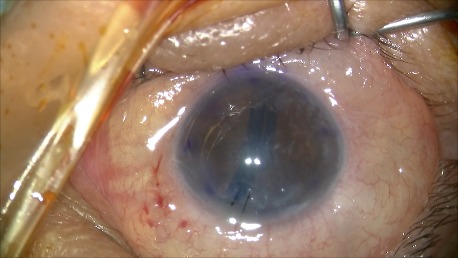
Double rolled graft in the stable anterior chamber, well oriented

**Fig. 2 D:**
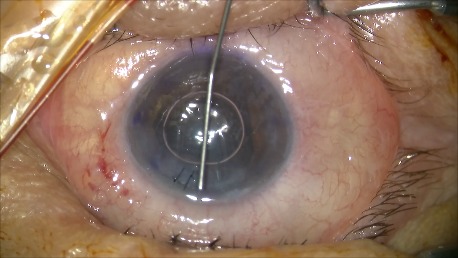
Unrolling and positioning the graft with the aid of an air bubble and tapping the cornea

**Fig. 2 E:**
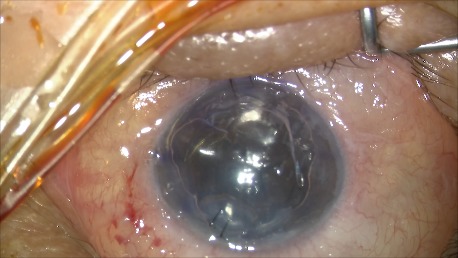
The graft is attached to the stroma, using air tamponade

Postoperatively, the patient received a bandage contact lens to protect the cornea until epithelial healing and topical treatment with antibiotic, steroids, lubricating eye drops, hypertonic solution. We used ciprofloxacin drops per 1 week and tobramycin/ dexamethasone combination four times a day, for 1 month. After the first month, dexamethasone drops were used three times a day for 2 months and were tapered by one drop at every 2 months, after that, dexamethasone was substituted with fluorometholone drops three times a day with progressive decreasing doses.

On postoperative week 1, the graft was attached but the corneal edema was significant and the visual acuity was decreased. Anterior segment OCT showed the attached endothelium graft with inferior peripheral folds (**Fig. 3 A**,**B**).

**Fig. 3 A:**
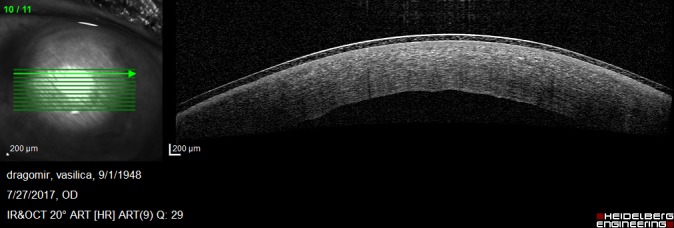
Anterior segment OCT at 1 week postoperatively shows irregular posterior corneal surface, increased central corneal thickness, attached endothelium graft

**Fig. 3 B:**
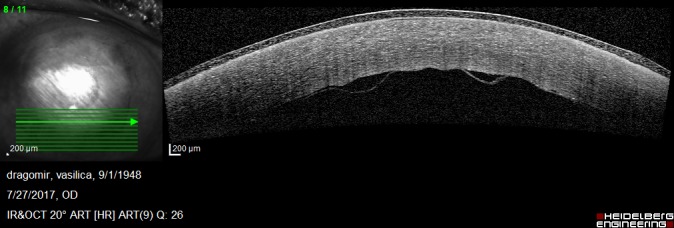
Anterior segment OCT at 1 week postoperatively shows inferior peripheral folds

On postoperative week 2, the corneal edema decreased and BCVA was 0.4, dilated fundus exam showed retinal angiosclerosis, and a good number of endothelial cells was found at cellular microscopy (**Fig. 4 A**-**C**).

**Fig. 4 A:**
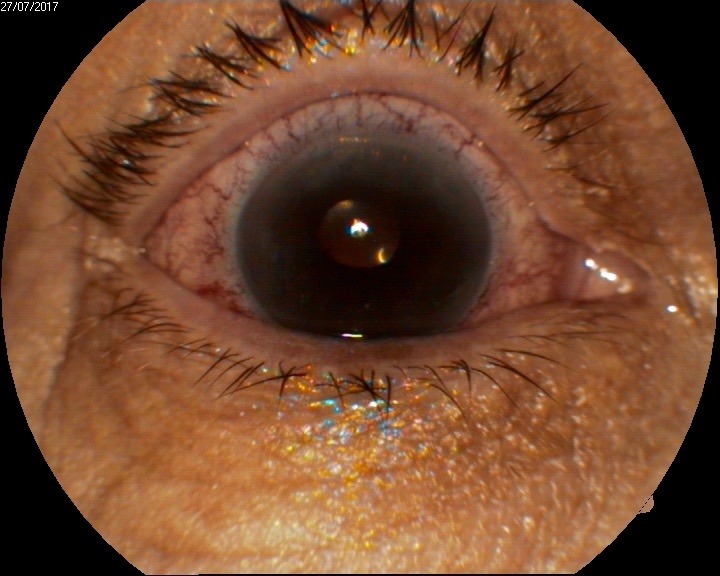
Anterior segment examination at 2 weeks shows clear cornea

**Fig. 4 B:**
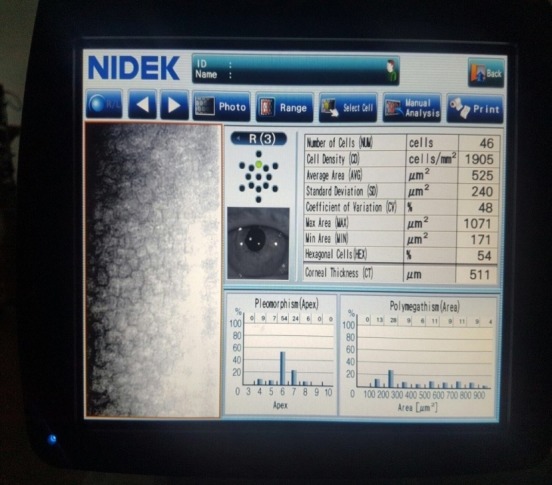
Endothelial cell density at 2 weeks is 1905 cells/ mm²

**Fig. 4 C:**
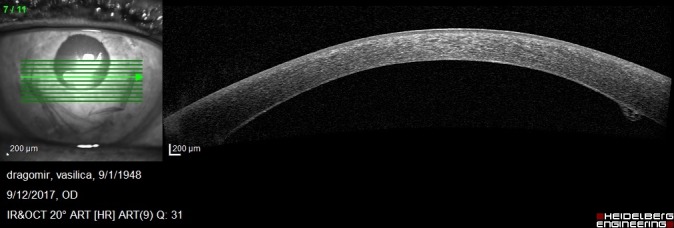
Anterior segment OCT at 2 weeks shows the attached endothelium

1 month postoperatively, BCVA was 0,6 and increased at 0,7 at 3 months after the surgical procedure. Good endothelial cells number and improved OCT aspects were observed: attached graft, decreasing central corneal thickness, stable peripheral folds (**Fig. 5 A**-**D**).

**Fig. 5 A:**
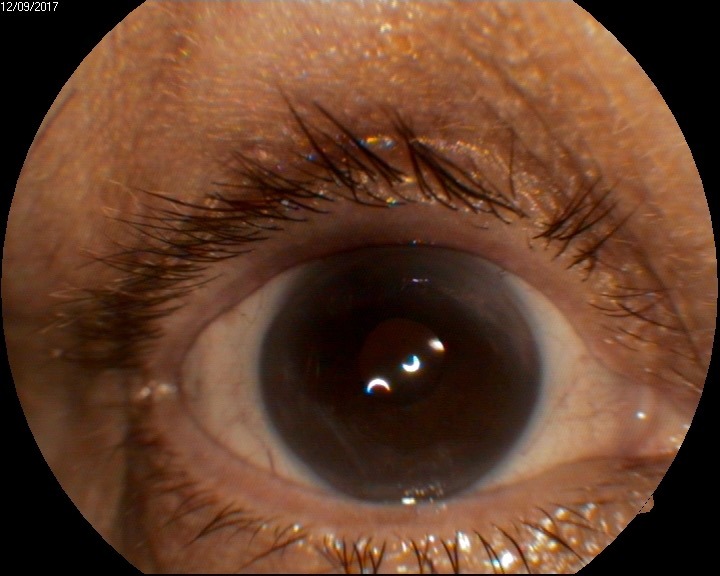
Anterior segment examination shows clear cornea

**Fig. 5 B:**
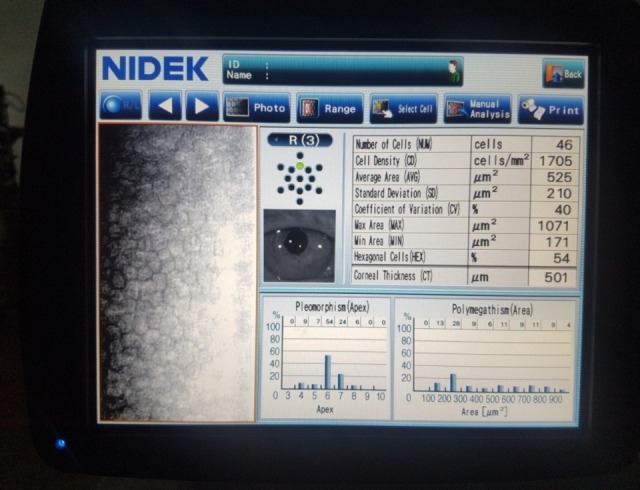
Endothelial cell density at 3 months is 1705 cells/ mm²

**Fig. 5 C:**
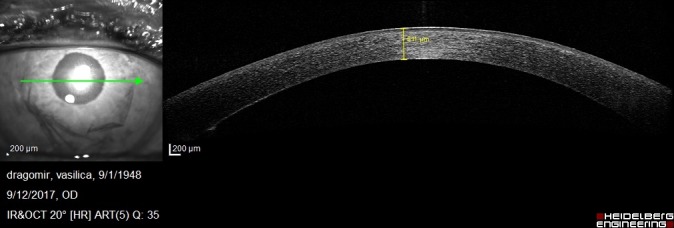
Anterior segment OCT shows the attached endothelium

**Fig. 5 D:**
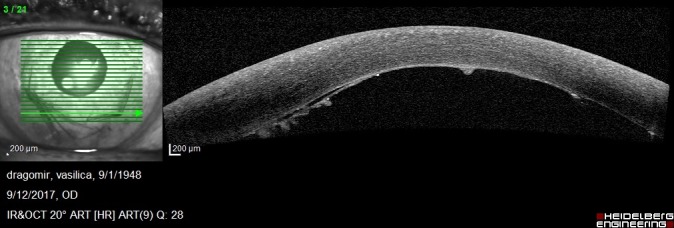
Anterior segment OCT shows stable inferior folds

## Conclusions

DMEK is an efficient alternative to penetrating keratoplasty, presenting many advantages in selected cases. The good BCVA obtained and the lower rejection risk makes DMEK a preferable technique for more and more surgeons. DMEK is a delicate, highly complex surgery so that it requires a longer learning curve to master standard reproducible techniques for corneal endothelium manipulation [**1**] but the learning curve does not correlate with the clinical outcome [**9**]. During a learning curve, an ideal DMEK candidate for endothelial failure due to Fuchs dystrophy should be pseudophakic, with no prior cataract surgery complications, no prior vitrectomy, nor glaucoma filtrating surgery [**7**] and have a good intraoperative visibility with or without desepitheliation. 

The good BCVA obtained in our patient postoperatively (0.1 preoperatively to 0.7 at 3 months after surgery) is encouraging for resolving similar cases with this technique. There are some disadvantages though: the poor availability of donors and the necessity of selecting the donor for an optimal result. 
